# Supplementary material for: Lateral Habenula 5-HT2C Receptor Function Is Altered by Acute and Chronic Nicotine Exposures
Source: Int J Mol Sci. 2021 Apr 30;22(9):4775. doi: 10.3390/ijms22094775 (PMC8124296; doi:10.3390/ijms22094775)
Supplement: Supplementary file 1 [file ijms-22-04775-s001.zip › ijms-1187739-supplementary.pdf]

International Journal of
Molecular Sciences

MDPI

Supplementary Materials:

Article

Lateral Habenula 5-HT_{2C} Receptor Function is Altered by Acute and Chronic Nicotine Exposures

Cristiano Bombardi [1], Francis Delicata [2], Claudio Tagliavia [1], Annamaria Grandis [1], Massimo Pierucci [2], Antonella Marino Gammazza[3], Maurizio Casarrubea [4], Philippe De Deurwaerdère [5] and Giuseppe Di Giovanni [2,6,*]

1 Department of Veterinary Medical Sciences, University of Bologna, Via Tolara di Sopra, 50, 40064 Ozzano dell'Emilia (BO), Italy; cristiano.bombardi@unibo.it (C.B.); claudio.tagliavia2@unibo.it (C.T.); annamaria.grandis@unibo.it; (A.G.)
2 Laboratory of Neurophysiology, Department of Physiology and Biochemistry, Faculty of Medicine and Surgery, University of Malta, Msida, MSD2080, Malta; francis.delicata.06@um.edu.mt (F.D.); Massimo.pierucci@um.edu.mt (M.P.); giuseppe.digiovanni@um.edu.mt (G.D.G.)
3 Section of Human Anatomy, Department of Biomedicine, Neuroscience and Advanced Diagnostics (BiND), University of Palermo, Palermo, Italy; antonella.marinogammazza@unipa.it (A.M.G.)
4 Laboratory of Behavioral Physiology, Human Physiology Section "Giuseppe Pagano", Department of Biomedicine, Neuroscience and Advanced Diagnostics (BiND), University of Palermo, Palermo, Italy; maurizio.casarrubea@unipa.it (M.C)
5 Centre National de la Recherche Scientifique (Unité Mixte de Recherche 5287); 146 rue Léo Saignat, B.P.281, F-33000 Bordeaux Cedex, France; philippe.de-deurwaerdere@u-bordeaux.fr (P.D.D)
6 School of Biosciences, Cardiff University, Cardiff, CF10 3AX, UK.
* Correspondence: giuseppe.digiovanni@um.edu.mt;digiovannig@cardiff.ac.uk; Tel.: +356-23402776

1. Results

1.1 Immunoperoxidase experiments

The distribution of the 5-HT_{2C}R immunoreactivity was particularly high in the NAcc, VTA and ST. These areas contained many immunostained cells distributed throughout their rostro-caudal extension. 5-HT_{2C}R immunoreactivity in these areas did not show any evident difference comparing the different groups (Supp Figures S1–S5; tables 2 main text).

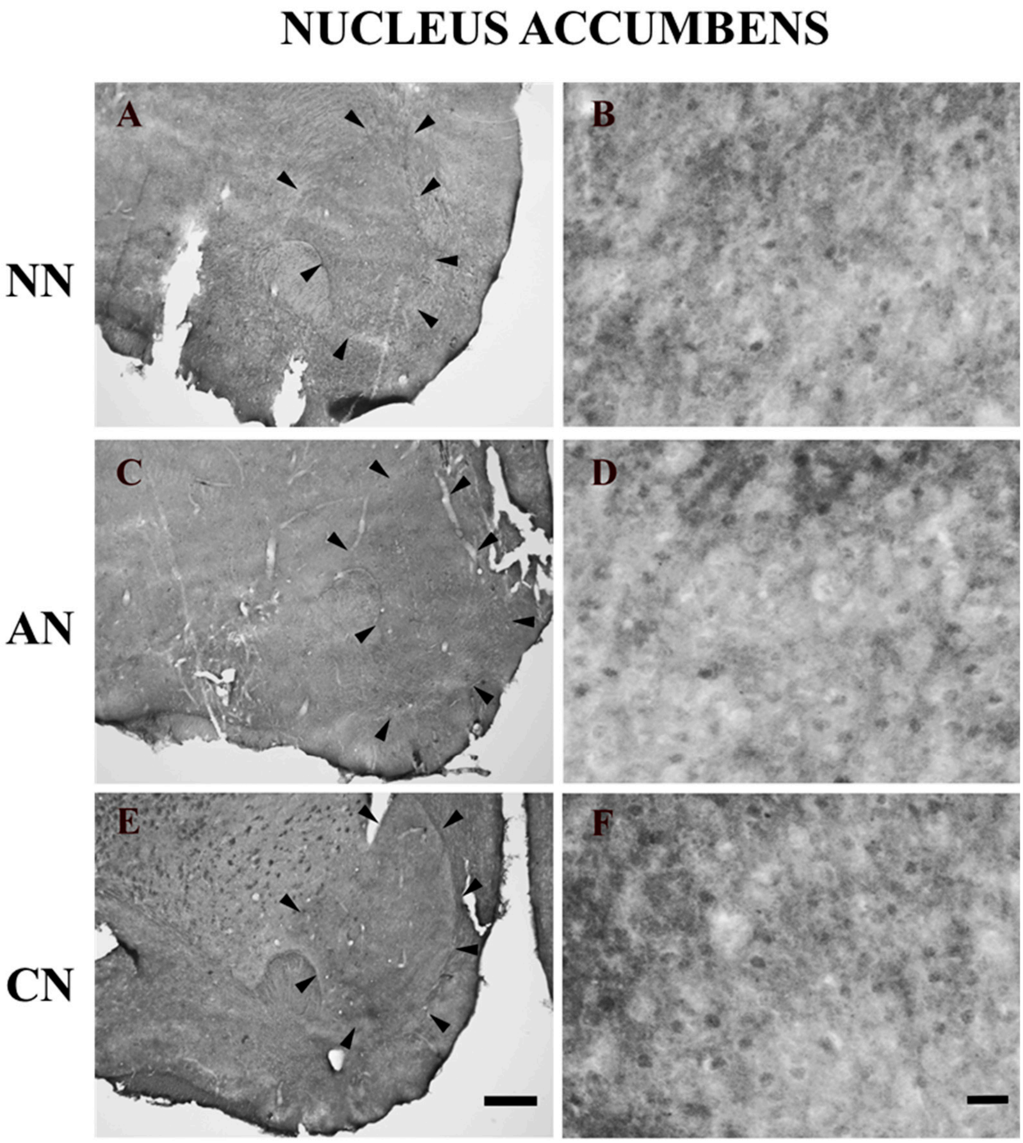

Supplementary Figure S1. Brightfield photomicrographs of coronal sections showing the distribution of 5-HT2C receptors (5-HT2CRs) immunoreactivity in the nucleus accumbens (bordered by arrowheads) of nicotine-naïve (NN; **A**,**B**), acute nicotine (AN; **C**,**D**), and chronic nicotine (CN; **E**,**F**) rats. Note that the immunostaining is similar in the different groups. See main text and tables 2 for explanations. Scale bar = 400 µm in **E** (applies to **A, C, E**) and 50 µm in **F** (applies to **B, D, F**). Naïve nicotine (NN, n=8), acute nicotine (AN, n=8) and chronic (CN, n=8).

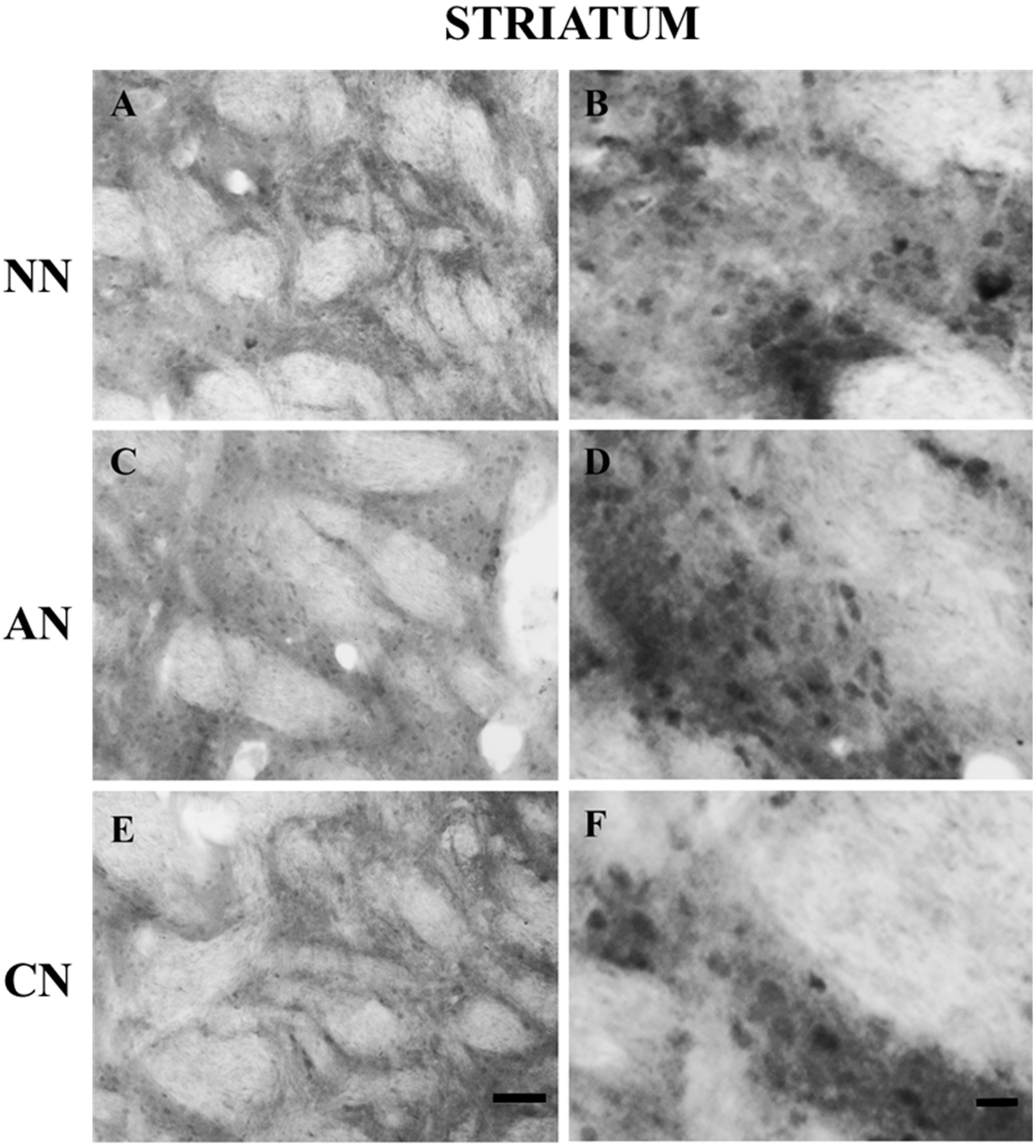

Supplementary Figure S2. Brightfield photomicrographs of coronal sections showing the distribution of 5-HT_{2C} receptors (5-HT_{2C}Rs) immunoreactivity in the nucleus accumbens (bordered by arrowheads) of nicotine-naïve (NN; **A**, **B**), acute nicotine (AN; **C**, **D**), and chronic nicotine (CN; **E**, **F**) rats. Note that the immunostaining is similar in the different groups. See main text and tables 2 for explanations. Scale bar = 150 μm in **E** (applies to **A, C, E**) and 3 μm in **F** (applies to **B, D, F**). Naïve nicotine (NN, n=8), acute nicotine (AN, n=8) and chronic (CN, n=8).

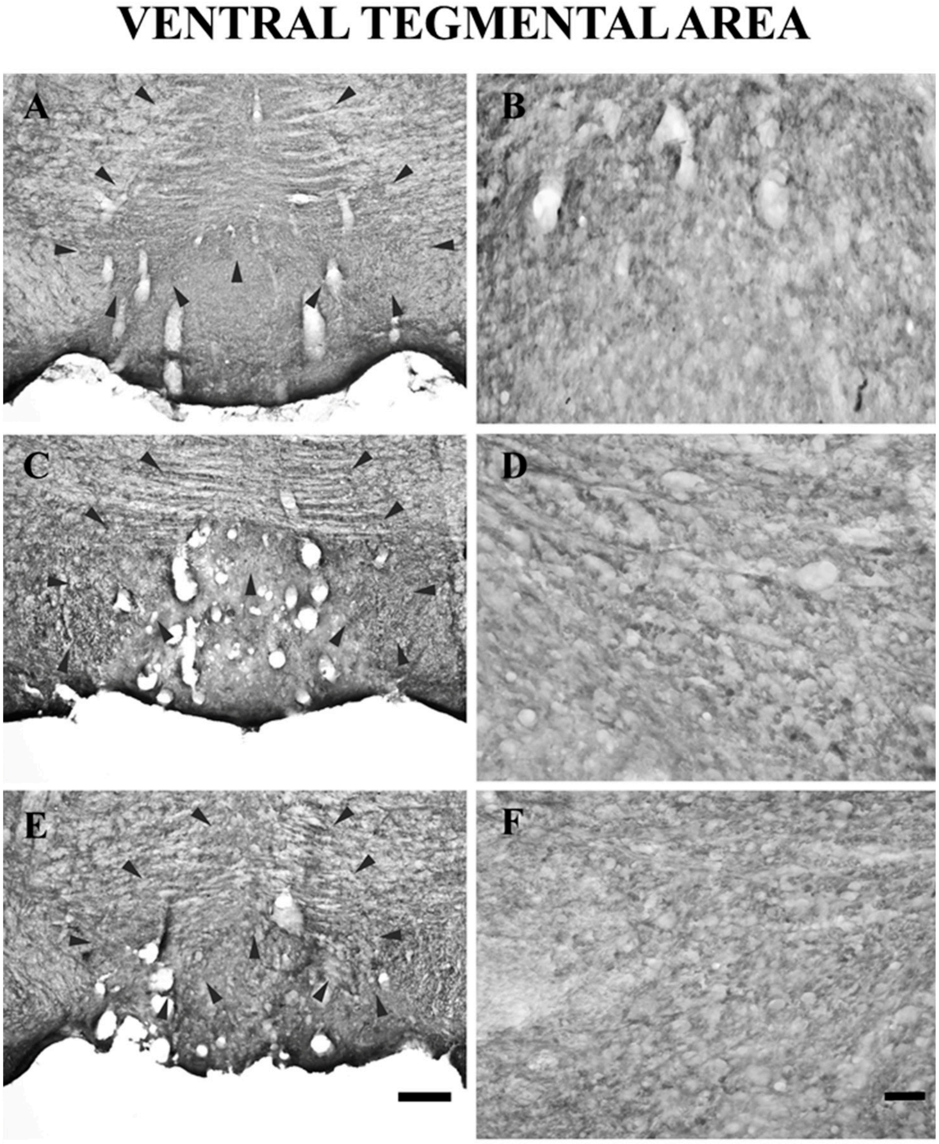

Supplementary Figure S3. Brightfield photomicrographs of coronal sections showing the distribution of 5-HT_{2C} receptors (5-HT_{2C}Rs) immunoreactivity in the ventral tegmental area (bordered by arrowheads) of nicotine-naïve (NN; **A**, **B**), acute nicotine (AN; **C**, **D**), and chronic nicotine (CN; **E**, **F**) rats. Note that the immunostaining is similar in the different groups. See main text and tables 2 for explanations. Scale bar = 200 μm in **E** (applies to **A, C, E**) and 50 μm in **F** (applies to **B, D, F**). Naïve nicotine (NN, n=8), acute nicotine (AN, n=8) and chronic (CN, n=8).

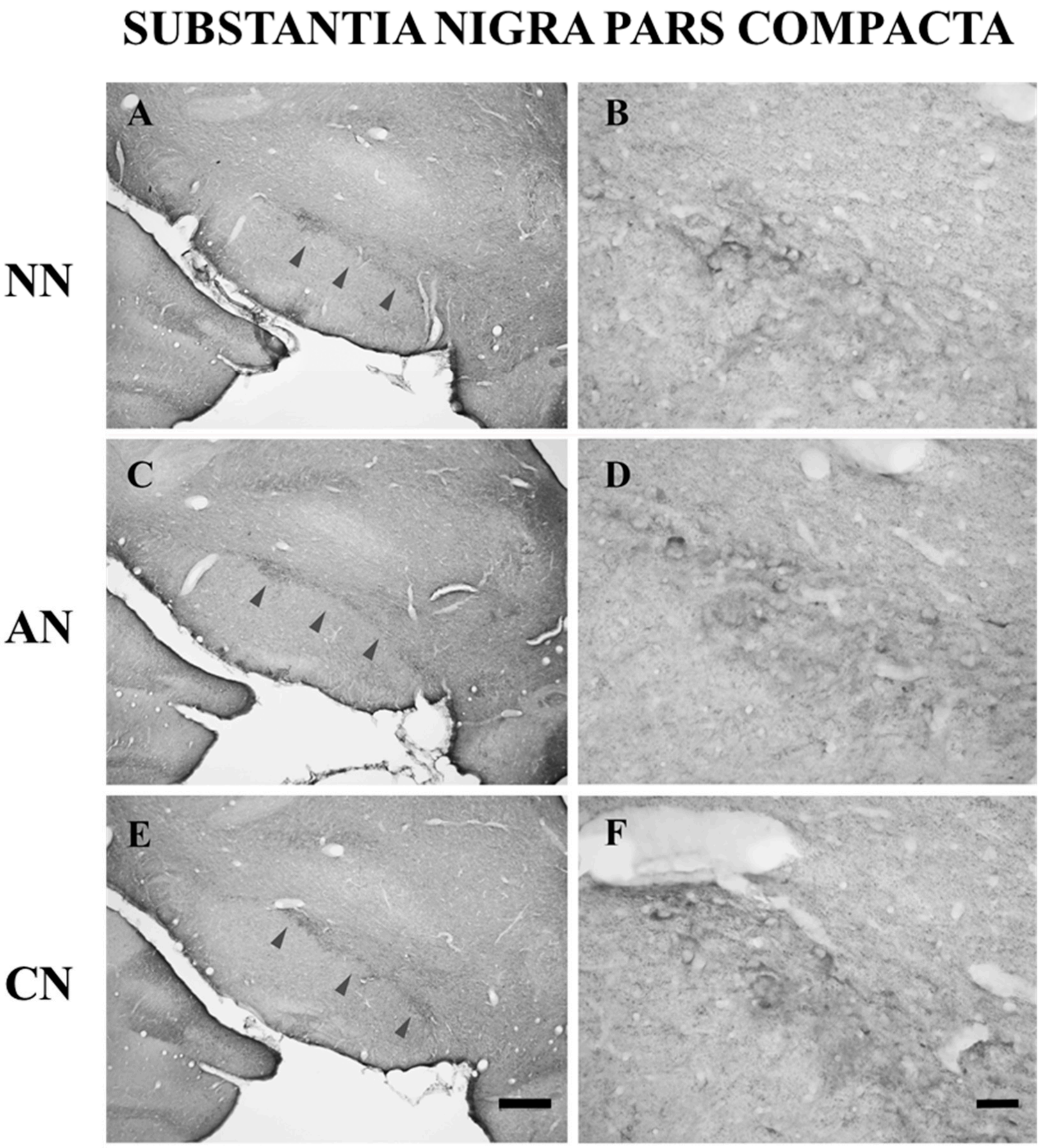

Supplementary Figure S4. Brightfield photomicrographs of coronal sections showing the distribution of 5-HT2C receptors (5-HT2CRs) immunoreactivity in the substantia nigra pars compacta (bordered by arrowheads) of nicotine-naïve (NN; **A**, **B**), acute nicotine (AN; **C**, **D**), and chronic nicotine (CN; **E**, **F**) rats. Note that the immunostaining is similar in the different groups. See main text and tables 2 for explanations. Scale bar = 400 μm in **E** (applies to **A, C, E**) and 50 μm in **F** (applies to **B, D, F**). Naïve nicotine (NN, n=8), acute nicotine (AN, n=8) and chronic (CN, n=8).

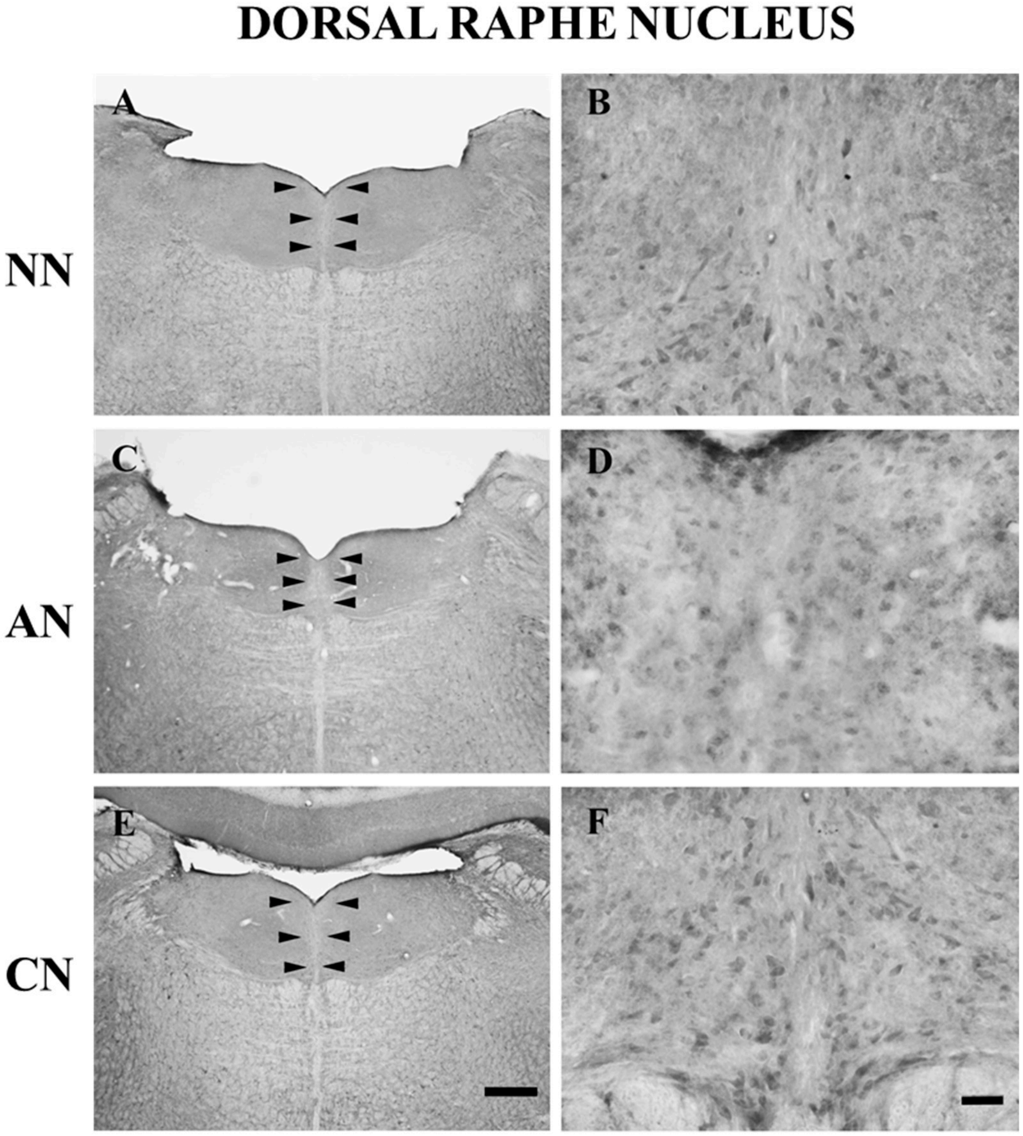

Supplementary Figure S5. Brightfield photomicrographs of coronal sections showing the distribution of 5-HT2C receptors (5-HT2CRs) immunoreactivity in the dorsal raphe nucleus (bordered by arrowheads) of nicotine-naïve (NN; **A**, **B**), acute nicotine (AN; **C**, **D**), and chronic nicotine (CN; **E**, **F**) rats. Note that the immunostaining is similar in the different groups. See main text and tables 2 for explanations. Scale bar = 400 µm in **E** (applies to **A, C, E**) and 40 µm in **F** (applies to **B, D, F**). Naïve nicotine (NN, n=8), acute nicotine (AN, n=8) and chronic (CN, n=8).

2.1 Materials and Methods

2.1 Histological Procedures and Immunocytochemistry

2.1.1 Animals and fixation

Twenty four rats (eight drug naive, eight acute nicotine, and eight chronic nicotine) were used in this study. The animals were deeply anesthetized with a mixture (4.0 ml/kg) of sodium pentobarbital (48 mg/kg) and chloral hydrate (40 mg/kg; intraperitoneally) and perfused intracardially using a peristaltic pump (flow rate 30-35 ml/min) as follows: 0.9% saline (+ 4°C) for 2 minutes, followed by a solution of 4% paraformaldehyde in 0.1 M sodium phosphate buffer, pH 7.4 (flow rate 10 ml/min) for 30 minutes. The brains were removed from the skull and postfixed in the final fixative for 2 to 4 hours. The brains were then cryoprotected in 30% sucrose solution in phosphate buffered saline (PBS), pH 7.4 at + 4° C for 48

hours and cut in the coronal plane at 30 μm section thickness on a freezing sliding microtome. The sections (1 in 5 series) were stored in 30% sucrose solution in PBS at –20°C (for immunohistochemical staining) or in 10% formalin at room temperature (for thionin staining) until processed.

2.1.2 Immunoperoxidase experiments

Immunoperoxidase experiments was performed on lateral habenula (LHb), nucleus accumbens (NAcc), ventral tegmental area (VTA), dorsal raphe nucleus (DRN), dentate gyrus (DG), substantia nigra pars compacta (SNpc), caudate-putamen (CP) and medial prefrontal cortex (mPFC). The free-floating coronal sections were washed three times (10 minutes each time) in 0.02 M PBS, pH 7.4. To eliminate endogenous peroxidase activity, the sections were treated with 1% H_2O_2 in H_2O for 15-30 min, and then rinsed 6 times in 0.02 PBS. To block non-specific binding, the sections were incubated in a solution containing 10% normal goat serum (Colorado Serum Co., Denver, CO, #CS 0922) and 0.3% Triton X-100 in 0.02MPBS for 2 hours at room temperature. Thereafter, the sections were incubated in a solution containing mouse anti-5-HT2CR monoclonal antibody (diluted 1:100; code sc-17797; Santa Cruz, CA, USA), 0.3% Triton X-100, and 1% normal goat serum for 48 h at 4°C. Following incubation in the primary antiserum, the sections were washed three times (10 min each) in 0.02M PBS containing 2% normal goat serum. Thereafter, the sections were incubated in a solution containing goat biotinylated anti-mouse (1:200, Vector, Burlingame, CA, BA-9200), 1% normal goat serum and 0.3% Triton X-100 in 0.02M PBS, pH 7.4 for 60 minutes at room temperature. The sections were washed three times for 10 min each in 0.02M PBS containing 2% normal goat serum and were then transferred to avidin–biotin complex (ABC kit Vectastain, PK-6100, Vector Laboratories, Burlingame, CA) for 45 min, and the immunoperoxidase reaction was developed by 3.3′-diaminobenzidine (DAB kit, SK-4100, Vector Laboratories, Burlingame, CA). After washing, the sections were mounted onto gelatin-coated slides, dried overnight at 37°C, defatted and intensified, according to Lewis et al. (1986), with OsO4 (0.005%, Electron Microscopy Sciences, #19130, Ft. Washington, PA) and thiocarbohydrazide (0.05%, Electron Microscopy Sciences, #21900), and coverslipped with Entellan (Merck, Darmstaldt, Germany).

2.1.3 Double immunofluorescence experiments

Double immunostaining was performed on LHb. Preliminary experiments were carried out to determine the optimal fixative mixture to be used in the double immunofluorescence methods. The final concentrations of primary antibodies were established by performing double immunofluorescence experiments using different dilution patterns. Whole, free-floating coronal sections were washed three times (10 minutes each) in PBS. To block non-specific binding, the sections were incubated in 10% normal goat serum (Colorado Serum Co., Denver, CO, #CS 0922) and 0.3% Triton X-100 in 0.02M PBS for 40 minutes at room temperature. Thereafter, the sections were washed three times (10 minutes each) in a solution containing 1% normal goat serum and 0.3% Triton X-100 in 0.02M PBS. For colocalization studies, the sections were incubated for 2 days at 4°C in a mixture of primary antibodies: mouse anti-5-HT2CR monoclonal antibody (diluted 1:100; code sc-17797; Santa Cruz, CA, USA) together rabbit anti-Protein Gene Product 9.5 (PGP 9.5) polyclonal antibody (diluted 1:100, AB1761, Millipore, Temecula, CA, USA), which were dissolved in 1% normal goat serum (Colorado Serum Co., Denver, CO, #CS 0922) and 0.3% Triton X-100 in 0.02M PBS. The sections were washed three times as described before and incubated overnight in a secondary antibody solution containing Alexa 488-conjugated goat anti-mouse IgG (1:400, #A11029, Molecular Probes, Leiden, the Netherlands), Alexa 594-conjugated goat anti-rabbit IgG (1:400, #A11012, Molecular Probes, Leiden, the Netherlands), 1% normal goat serum and 0.3% Triton X-100 in 0.02M PBS. The sections were then washed with 0.02M PBS and mounted on glass microscope slides (Super Frost Plus, Menzel-Glaser #J1800AMNZ), dried, and coverslipped using Vectashield Mounting Medium (#H-1000, Vector Laboratories, Burlingame, CA).

2.1.4 Specificity of antibodies

Mouse monoclonal antibody anti-5-$HT_{2C}R$ is directed against amino acids 374-458 of the C-terminus of 5-$HT_{2C}R$ of Human origin. The specificity of this antibody has previously been determined using western blot and immunohistochemical analyses (Morán et al., 2008; Anastasio et al., 2010; Nocjar et al., 2015). In the present experiments, control sections incubated without the primary antibody resulted in a complete disappearance of stained profiles. The specificity of the rabbit polyclonal antibody directed against PGP 9.5 (AB1761, Millipore) was determined in conducted by the manufacturer. The omission as well as the replaced of the secondary antibodies with inappropriate secondary antibodies resulted in elimination of all immunoistochemical staining.

2.1.5 Thionin staining

To help identify the boundaries of the LHb, NAcc, VTA, DRN, DG, SNpc, ST and mPFC, sections adjacent to immunolabelled sections were stained with thionin as follows. Sections were taken out of the 10% formaldehyde solution, mounted on gelatin-coated slides and dried overnight at 37 ◦C. Sections were defatted 1 h in a mixture of chloroform/ethanol 100% (1:1), and then rehydrated through a graded series of ethanol,2×2 min in 100% ethanol, 2 min in 96% ethanol, 2 min in 70% ethanol, 2 min in50% ethanol, 2 min in dH2O, and stained 30 s in a 0.125% thionin (Fisher Scientific) solution, dehydrated and coverslipped with Entellan (Merck, Darmstaldt, Germany).

2.1.6 Analysis of sections

2.1.6.1 Immunoperoxidase experiments

Sections were analyzed using a Leica DMRB microscope. Brightfield images were acquired by means of a Polaroid DMC digital camera (Polaroid Corporation, Cambridge, MA, USA) and DMC 2 software. Contrast and brightness were adjusted to reflect the appearance of the labeling seen through the microscope using Adobe Photoshop CS3 Extended 10.0 software (Adobe Systems, San Jose, CA). To calculate the density of 5-HT2CR-IR neurons, immunostained somata were plotted bilaterally throughout the LHb, NAcc, VTA, DRN, DG, SNpc, ST and mPFC with a computer-aided digitizing system (Accustage 5.1, St. Shoreview, MN). The boundaries of the different brain areas was drawn from the adjacent thionin-stained sections using a stereo microscope equipped with drawing tube. The outlines were superimposed on computer generating plots using Corel Draw X3 (Corel corporation, Ottawa, Ontario, Canada). The area measurements were done from the line drawings by using AxioVision Rel.4.8 (Zeiss). The density of immunostained neurons was calculated as number of somata/mm^2 in each section separately. For each rat the left and right LHb, NAcc, VTA, DRN, DG (polymorphic cell layer), SNpc, CP and mPFC (layers V and VI) located in 5 sections were analyzed. Below we report the approximate rostrocaudal distance from the bregma (according to the atlas of Paxinos and Watson, 1998) for each of the 5 analyzed sections: LHb (-3.14 mm; -3.30 mm; -3.60 mm; -3.80 mm; -4.16 mm), NAcc (+2.70 mm, +2.20 mm, +1.60 mm, +1.20 mm, +1.00 mm), VTA (-5.30 mm; -5.60 mm; -5.80 mm; -6.04 mm; -6.30 mm), DRN (-7.80 mm; -8.00 mm; -8.30 mm; -8.80 mm; -9.16 mm), DG (-2.80 mm; -3.30 mm; -3.80 mm; -4.30 mm; -4.80 mm), SNpc (-5.20 mm; -5.30 mm; -5.60 mm; -5.80 mm; - 6.04 mm), CP (+0.20 mm; -0.30 mm; -0.92 mm; -1.60 mm; -2.30 mm) and mPFC (+5.20 mm; +4.70 mm; + 4.20 mm; +3.70 mm; +3.20 mm). The neuronal counts are expressed as the mean number of somata/mm^2 ± standard deviation and the data from the drug naive, acute nicotine, chronic nicotine and chronic nicotine withdrawal rats were compared. The Student's t-test was used for comparing the statistical differences between drug naive, acute nicotine, chronic nicotine and chronic nicotine withdrawal rats, with a significance level at $P < 0.05$. To determine neuronal densities only somata with an evident immunostaining were considered. Lightly stained neurons, or neurons with an immunoreactivity difficult to distinguish from that of the surrounding neuropil, have not been counted. Data concerning the percentage of the image covered by 5-$HT_{2C}R$ immunoreactivity were obtained using

the automatic threshold algorithm of ImageJ (version IJ 1.46r downloaded from http://imagej.nih.gov/ij/download.html). For this analysis images were taken using a Leica DMRB microscope using identical acquisition parameters from drug naive, acute nicotine and chronic nicotine. For each animal, 5 sections were analyzed. All data are given as mean ± standard deviation (SD) and differences were evaluated using the Student's t-test, with a significance level of $p < 0.05$.

2.1.6.2 Double immunofluorescence experiments

Double immunofluorescence sections were analyzed with a Nikon H550L (Nikon Instruments, Japan) equipped with the appropriate filter cubes for immunofluorescence. We used the FITC filter for Alexa 488 (Ex 465–495; DM 505; BA 515–555) and the TRITC filter for Alexa 594 (EX 540/25; DM 565; BA 605–655). For immunofluorescence analysis, the neurons were first located by the presence of a fluorophore which labeled one antigen, and the filter was then switched to a fluorophore specific for a different wavelength to determine whether or not the neuron was labeled for a second antigen. In this way, the proportions of neurons labeled for pairs of antigens or for a single antigen were determined. Bilateral colocalization studies were assessed at the various rostrocaudal levels of LHb. In particular, counts of single- and double-labeled neurons were carried out in five non-consecutive sections obtained from the right and left LHb of each rat (approximate rostrocaudal distance from the bregma, according to the atlas of Paxinos and Watson, 1998: -3.14 mm; -3.30 mm; -3.60 mm; -3.80 mm; -4.16 mm). Differences between drug naive, acute nicotine, chronic nicotine and chronic nicotine withdrawal rats were evaluated using Student's t-test, with a significance level at $P < 0.05$. The images were recorded using a Nikon-Qi1Mc photocamera (Nikon Instruments, Japan) and Nikon Elements Version 4.10 software. The contrast and brightness of the figures were adjusted to reflect the appearance of the labelling seen through the microscope using Adobe Photoshop CS3 Extended 10.0 software (Adobe Systems, San Jose, CA).

2. Electrophysiological Recordings

2.2.1 Extracellular Single-Unit Recordings

Rats were anesthetized with chloral hydrate (400 mg/kg, i.p., Sigma-Aldrich, UK) and anesthesia was maintained with a continuous intravenous (i.v.) infusion of chloral hydrate (8% *w*/*v*; 8 mL/h). Standard single-unit extracellular recording of LHb neurons was performed as described previously. Micropipettes (4–7 MΩ resistance) were positioned in the LHb (set at a 10° angle, 3.4–3.8 mm anteroposterior from bregma, 1.4–1.8 mm mediolateral from the midline, 4–5 mm dorsoventral from the cortical surface). Signal acquisition was made with a micro1401 CED laboratory interface connected to Spike2 v7.4 (Cambridge Electronic Design, Cambridge, UK) and a Neurolog amplifier and filtering system (Digitimer Ltd. UK; 10k amplification, bandpass filter set at 0.5–5 kHz). The location of the recording site was confirmed with histological analysis.

Pharmacological Treatments

Acute nicotine treatment involved a single intraperitoneal dose of nicotine (2 mg/kg, i.p.) one hour before brain extraction or neuronal recording. Chronic nicotine treatment involved three daily doses of 2 mg/kg, i.p., for fourteen days, followed by an additional dose one hour before brain extraction or neuronal recording. Ro 60-0175 was administered as a cumulative dose of 640 µg/kg, given as eight separate 100 µL doses (5, 5, 10, 20, 40, 80, 160 and 320 µg/kg), two minutes apart.

The average firing rate ($FR_{baseline}$) from the initial 10 min baseline recording was used to normalize the data for subsequent time points (FR_x), i.e., the 2 min following each of the eight cumulative agonist doses. Firing activity was expressed as the percentage change in firing for each time point with the baseline firing: $\left(\frac{FR_x - FR_{baseline}}{FR_{baseline}}\right) \times 100$. If two-time points recorded a 20% increase or a 20% decrease in neuronal activity, or none of the above, the LHb neuron was classified belonging to the increase, decrease, or null group, respectively, according to published criteria (Delicata et al., 2018).

Statistical Analysis

The data report the mean ± SEM for each group if any. One-way ANOVA with repeated measures was performed separately for each treatment group compared to their respective controls. It was followed by Tukey post-hoc test (if required). A significant difference was considered when $p < 0.05$.

References

Delicata, F., Bombardi, C., Pierucci, M., Di Maio, R., De Deurwaerdère, P., and Di Giovanni, G. (2018). Preferential modulation of the lateral habenula activity by serotonin-2A rather than-2C receptors: Electrophysiological and neuroanatomical evidence. *CNS neuroscience & therapeutics* 24**,** 721-733.

Paxinos, G., Watson, C. The Rat Brain in Stereotaxic Coordinates, Fourth edition, Academic Press, New York, 1998.
